# Finding Traceability Granularity Influencing Factors Using Rough Set Method: An Empirical Analysis of Vegetable Companies in Tianjin City, China

**DOI:** 10.3390/foods12112124

**Published:** 2023-05-24

**Authors:** Jianping Qian, Jiali Li, Bojian Geng, Cunkun Chen, Jianjin Wu, Haiyan Li

**Affiliations:** 1 Key Laboratory of Agricultural Remote Sensing (AGRIRS), Ministry of Agriculture and Rural Affairs/Institute of Agricultural Resources and Regional Planning, Chinese Academy of Agricultural Sciences, Beijing 100081, China; lijiali@cau.edu.cn; 2 Yangtze River Delta Intelligent Agriculture Research Institute of Chinese Academy of Agricultural Sciences, Suzhou 215331, China; gbj2859601614@163.com; 3 Institute of Agricultural Products Preservation and Processing Technology (National Engineering Technology Research Center for Preservation of Agriculture Product), Tianjin Academy of Agricultural Sciences/Key Laboratory of Postharvest Physiology and Storage of Agricultural Products, Ministry of Agriculture and Rural Affairs, Tianjin 300384, China; chencunkun@126.com; 4 Tianjin Agricultural Development Service Center, Tianjin 300061, China; 13602062024@139.com (J.W.); lihaiyan_1969@126.com (H.L.)

**Keywords:** traceability granularity, influence factors, rough set, empirical analysis, vegetable companies

## Abstract

The effectiveness evaluation of the traceability system (TS) is a tool for enterprises to achieve the required traceability level. It plays an important role not only for planning system implementation before development but also for analyzing system performance once the system is in use. In the present work, we evaluate traceability granularity using a comprehensive and quantifiable model and try to find its influencing factors via an empirical analysis with 80 vegetable companies in Tianjin, China. We collect granularity indicators mostly through the TS platform to ensure the objectivity of the data and use the TS granularity model to evaluate the granularity score. The results show that there is an obvious imbalance in the distribution of companies as a function of score. The number of companies (21) scoring in the range (50,60) exceeded the number in the other score ranges. Furthermore, the influencing factors on traceability granularity were analyzed using a rough set method based on nine factors pre-selected using a published method. The results show that the factor “number of TS operation staff” is deleted because it is unimportant. The remaining factors rank according to importance as follows: Expected revenue > Supply chain (SC) integration degree > Cognition of TS > Certification system > Company sales > Informationization management level > System maintenance investment > Manager education level. Based on these results, the corresponding implications are given with the goal of (i) establishing the market mechanism of high price with high quality, (ii) increasing government investment for constructing the TS, and (iii) enhancing the organization of SC companies.

## 1. Introduction

With ever more attention being devoted to the topic of food safety, traceability is looked at as an effective method to ensure food safety and quality and to reduce the costs associated with recalls [1,2,3,4,5]. Traceability is defined in international standards, legislation, and even dictionaries [6,7]. Olsen and Borit offered a new definition, namely, the ability to access any or all information relating to that which is under consideration, throughout its entire life cycle, by means of recorded identifications [8].

Driven by food safety and quality as well as by regulatory, social, economic, and technological concerns, mandatory or voluntary traceability systems are now being enforced worldwide [9,10,11,12]. Several systems of government supervision, like the EU Rapid Alert System for Food and Feed (RASFF), the Food Modernization and Safety Act (USA), and the National Agriculture and Food Traceability System (Canada), have been implemented [13,14]. To improve company supply chain management (SCM), research has focused on state traceability systems and their application for satisfying the various requirements of agro-food or food quality, such as for vegetables [15,16], fruits [17,18,19], olive oil [20], aquaculture [21], meat [22], or beef [23,24].

It is very important to measure the degree of traceability when working with a widely applied TS. Such measurements play an important part not only in system implementation plans before deployment but also in analyzing system performance after the system is in use [25]. Precision, breadth, and depth were the early metrics for TS [26]. Precision reflects the degree of assurance with which the TS can pinpoint the movement or characteristics of a particular food product. Breadth describes the amount of information the TS records, and depth describes how far back or forward the TS tracks. Next, granularity was defined to reflect the size and number of product batches [27]. Finer traceability granularity means increasingly detailed information about a product and allows for recalls to be done on a more detailed and range-limited level [28]. In addition, other metrics are used to measure TSs, such as purity in horticultural pack-house-processing transformations [29] and capability, rapidity, and accuracy in fish-processing plants [30].

Targeting a comprehensive and quantifiable TS, Qian et al. proposed a novel traceability-granularity model for agro-food [31]. The model includes a comprehensive evaluation index that combines precision, breadth, and depth with a quantifiable evaluation model to measure TS level. The model was applied in preliminary tests to two companies in the wheat-flour supply chain. However, the preliminary tests may result from three limitations: (i) ignoring the company’s characteristics; (ii) limiting the survey samples; and (iii) confusing internal traceability with chain traceability. To overcome these shortcomings and resolve these puzzles, the traceability-granularity model was validated using data generated by 80 vegetable companies. Moreover, the influencing factors were analyzed based on a rough set to find the driving forces causing differences in traceability and granularity.

In this paper, Section 2 introduces the granularity-evaluation model, which is based on previous research. Section 3 describes the materials and methods, and Section 4 presents the results of the granularity evaluation and analyzes the factors that influence it. The main conclusions and policy suggestions are presented in Section 5.

## 2. Granularity-Evaluation Model and Influence Factors

### 2.1. Granularity-Evaluation Model to Measure Traceability

Defining and evaluating the performance of the TS is an important means to measure the differentiation and implementation effects of the traceability system. Qian et al. developed a traceability-granularity model to measure agro-food TS [31]. The model was constructed by using a two-layer index system, in which the first layer includes mainly factors such as precision, breadth, and depth, and the second layer includes seven indicator sub-factors: external trace units, internal flow units, identification unit (IU) conversion, information collection content, information update frequency, forward-tracking distance, and backward-tracking distance. The score of each indicator is rated by some 20 experts. The weight value of a granularity evaluation indicator is calculated by an analytic hierarchy process. The weights of the seven indicators were 0.1985, 0.1141, 0.0872, 0.1870, 0.1248, 0.1442, and 0.1442, respectively, as shown in Table 1.

We use a weighted sum to evaluate the traceability granularity:

(1) $$S=20\left(\sum_{i=1}^{n}s_{i}w_{i}\right)$$

In this formula, *n* is the number of indicators, $s_{i}$ is the score of indicator *i*, and $w_{i}$ is the weight of indicator *i*. Considering the evaluation involves a five-score system, the overall evaluation scores are extended by a factor of 20; therefore, the total evaluation score is 100, which increases the discrimination.

The evaluation score represents a comprehensive result. The higher the evaluation score, the higher the traceability granularity.

### 2.2. Selection of Factors That Influence Traceability

Recently, research has focused on the factors that influence the motivation to implement traceability, such as expected revenue, government policy, market requirements, authentication systems, company characteristics, human resources, and so on [32,33,34,35]. These main influencing factors are listed in Table 2.

From the factors found in this selection of literature, we selected nine and rejected two. Considering the TS was established by the government and applied according to the actual environment in which each company evolved, the factor of government policy can be seen as a similarity. Most vegetable companies deal with the local market or processing companies, so the market requirement is the same for most companies. Thus, we neglect the factors of government policy and market requirements in this research. Nine factors were selected for analyzing the factors that influence TS granularity. First, we conducted a preliminary investigation of 15 companies to determine the range of these factors. The main factors and the associated range are listed below.

(1) Expected revenue

The success of applying the TS to companies depends strongly on net revenue. When the expected revenue exceeds the investment, the company is motivated to use the TS. In this research, expected revenue is divided into five ranges: <0%, 0–5%, 5–10%, 10–15%, 15–20%, and >20%.

(2) Certification system

To a certain extent, quality certification reflects the importance that the company places on quality and safety. Certification systems include ISO 9000 certification, the Good Agriculture Practice, the Hazard Analysis and Critical Control Point, and other certifications, such as the Green Food Certification or the Organic Food Certification.

(3) Degree of SC integration

The stages of the vegetable supply chain include planting, processing, wholesale, and retail. The degree of SC integration indicates the degree to which SC stages are incorporated into company procedures, be they internal to the company or between companies. A higher degree of integration corresponds to a higher degree of SC integration, and vice versa.

(4) Education level of managers

Management desire plays an important part in implementing a TS. The education level of managers is related to the long-term viability of the TS. The education level of managers is divided into five categories: non-high school graduate, high school degree, college degree, master’s degree, and doctoral degree.

(5) Company sales

Company sales are an important factor. In this paper, sales in 2015 serve as an indicator of company size. The 80 companies were divided into five ranges of sales: <5 million yuan, 5–10 million yuan, 10–30 million yuan, 30–50 million yuan, and >50 million yuan.

(6) Number of TS operation staff

Human resources impact TS performance. Here, the ratio of TS operation staff to regular staff is used as the human resources indicator.

(7) Level of information management

Information technology and management are key parts of implementing a TS. Equipment such as computers and barcode printers is necessary. Here, we use the number of computers per 100 people as an indicator of the level of information management.

(8) Cognition of TS

Although TS has spread in China in recent years, the cognition of TS by company managers is a step-by-step process. We evaluate the cognition of TS by asking company managers, “What is TS?”, “Which benefits can TS bring?”, “What is the main technology used to implement TS?”, and “Do you plan to use TS in your company?”

(9) Investment in system maintenance

Although system development is supported by the government, companies are expected to invest in system maintenance. To some extent, system-maintenance investment embodies the desire to sustainably use TS. To reflect this, we use the ratio of system-maintenance investment to company sales as an indicator.

## 3. Materials and Method

### 3.1. Study-Case Overview

**Figure 1:**
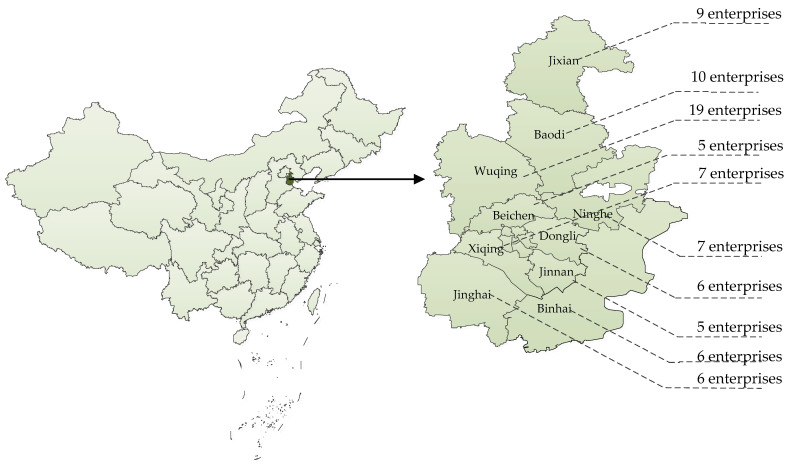
Study case position and survey company distribution in different counties in Tianjin city.

The study zone is in Tianjin city, which is one of the four municipalities under direct control of the Central Government of China (Figure 1). The “rest-assured vegetable action plan” was started in 2012 to ensure food safety. The goal was to construct 25,000 ha of pollution-free land in four years and produce 2.4 million tons of high-quality vegetables each year, capable of meeting the demand of the whole city. As of December 2016, 243 bases have joined this plan.

**Figure 2:**
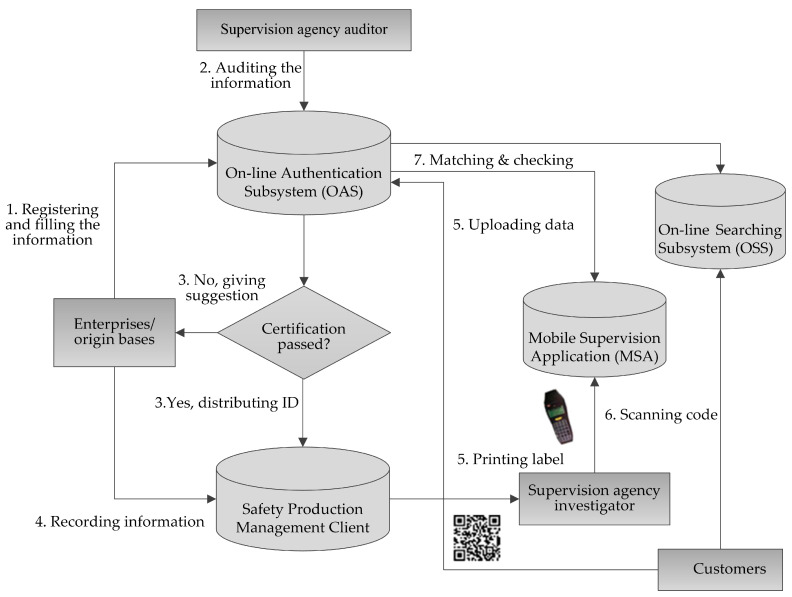
TS application processing with five subsystems.

To implement the plan, the TS was made mandatory. The TS includes four parts: the on-line authentication subsystem (OAS), the safety production management client (SMC), the mobile mobile application (MSA), and the on-line searching subsystem (OSS). Figure 2 shows a supervision and traceability flow framework with information and communication technologies. An OAS was applied by a supervisory agency to implement the origin base and its product authentication. The SMC was applied by the authorized origin base for information management and to print two-dimensional barcode labels with an authorized identification. The MSA was applied by the supervisory agency to check the authentication information in real time and implement on-scene communication with OAS. The OSS was used to implement the traceability information query.

We selected 80 vegetable companies in Tianjin city for this study. Although implementing traceability is mandatory, the performance level differs according to the situation of each company and their subjective desires. In other words, basic information and information batches were required from each company and were recorded to search for identification patterns, information content, and so on. This approach leads to differences in traceability granularity.

### 3.2. Data Collection to Evaluate Granularity

The TS platform aggregates a variety of detailed enterprise information, traceability information, and more. The granularity evaluation model requires seven indicators. The information can be collected via the OSS of the TS platform. When a traceability code is input into the system, the traceability information, including external trace units, simple information content, information-update frequency, forward-tracking distance, and backward-tracking distance, is displayed on the interface. Considering company trade secrets, customers are provided only partial information. Therefore, information related to internal flow units, IU conversion, and detailed information content is obtained from the SMC.

### 3.3. Characteristics of Companies Surveyed

**Figure 3:**
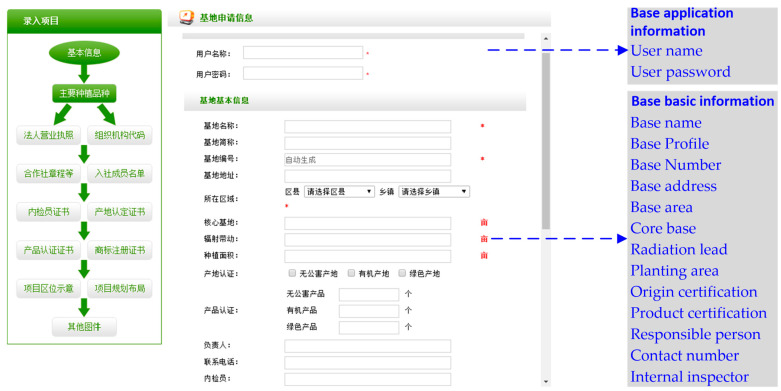
Obtaining influencing factor information in the enterprise registration interface on OAS.

With the TS used in Tianjin, information on nine factors that influence the TS’s success is available from the company registration interface on the OAS, as depicted in Figure 3. Eighty company characteristics are classified according to these influencing factors. As shown in Table 3, companies with expected revenue of 0–5% and 5–10% account for 30% and 46.25% of the sample, respectively. Thus, most of the companies surveyed clearly did not expect much revenue from implementing the TS. Other certifications, such as a certification of non-pollution (53.75%), dominate the sample of companies. Considering the vegetable-planting characteristics, most of the companies have either a medium or low degree of SC integration (15% and 47.5%, respectively). A few companies have a high or very high degree of SC integration because of a connection with a vegetable processing chain, a vegetable distribution chain, or even vegetable sales. For the managers, 60% have a college education. Only a few managers have no high school degree or a doctoral degree (5% and 2.5%, respectively). For annual company sales, 78.75% of the companies had less than 30 million yuan in 2015. For leadership in the vegetable industry, most companies have one designated TS employee, although some have none. The level of information management differs between companies, with most falling into the categories of medium and low. Finally, based on the survey of company managers, cognition of TS is mostly medium or high (50% and 26.25%, respectively). Thus, over half of the companies invest only 1–3% of sales in system maintenance.

### 3.4. Rough Set Method

Rough set theory can be used to identify and evaluate the dependence of data with the premise of retaining key information, revealing the importance of the condition attribute in determining the decision attribute, and removing redundant or unimportant condition attributes [49]. Details on rough set theory are available in the literature [50,51]. The rough set method has three parts: setting up the initial decision table, data preprocessing, and knowledge reduction [52]. The basic concepts are outlined below.

#### 3.4.1. Information System

Let S=(U,A,V,f) be an information system, where *U* is a nonempty finite universe; in this case, the 80 companies in the sample. *A* is a nonempty finite set consisting of *C* and *D*, which are the condition- and decision-attribute sets, respectively. In this study, the condition-attribute set consists of nine factors, and the decision-attribute set is the TS granularity grade. The TS granularity scores are divided into five grades that increment by 20. $V=\underset{a\in A}{\cup }V_{a}$, where *V_a_* is the numerical range of attribute a. f:U×A→V is the information function. The information system *S* = (*U*, *A*) is also called the decision table.

#### 3.4.2. Equivalence Relation

Let *R* be an equivalence relation in *U*. Each nonempty subset R⊂A determines an indiscernibility relation *IND(R)* that divides *U* into *k* categories: *X*_1_, *X*_2_, *X*_3_,..., *X_k_*. Each category represents a company from the sample of companies being investigated.

#### 3.4.3. Approximations and Positive Region

We construct lower and upper approximations to define the degree of approximation of each attribute. The lower approximation *R_(X)_*, also called the positive region of *X*, is denoted *POS_R_(X)*. *R_(X)_* is the certain element set classified as R⊂A, which is the maximum definition including *X*. ${\bar{R}}_{\left(X\right)}$ is the uncertain element set, which is the minimal definition including *X*.

#### 3.4.4. Attribute Importance

In information system *S*, the decision table is reduced according to the importance of the decision attribute. To start, to define the degree $\gamma_{C}\left(D\right)$ of dependency of each attribute, we calculate the ratio of the positive region element number of each attribute *|POS_C_(D)|* to the number of samples *|U|*. The equation is as follows:

(2) $$\gamma_{C}\left(D\right)=\frac{\left|{POS}_{C}\left(D\right)\right|}{\left|U\right|}$$

Next, we calculate the importance ${Sig}_{D}\left(C_{i}\right)$ of each condition attribute, where

(3) $${Sig}_{D}\left(C_{i}\right)=\gamma_{C}\left(D\right)-\gamma_{C-C_{i}}\left(D\right)$$

## 4. Results Analysis

### 4.1. Comparison of Granularity

**Figure 4:**
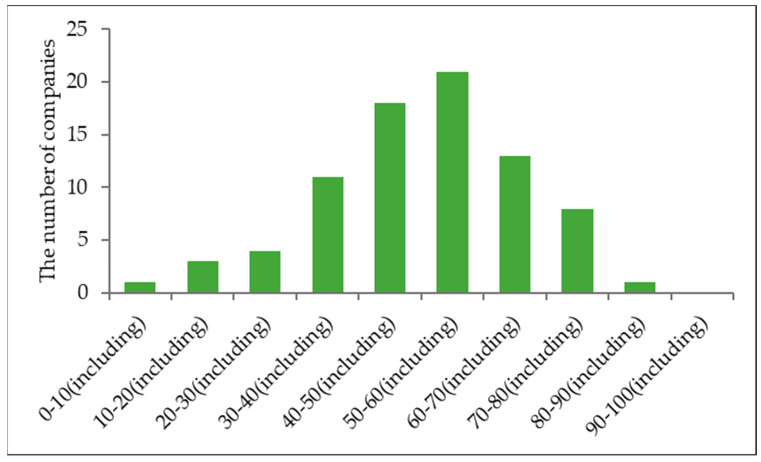
Granularity scores of the 80 investigated enterprises.

We use the model to evaluate TS granularity. The traceability granularity of the 80 companies is graded in score increments of 10, as shown in Figure 4.

Figure 4 shows that the distribution of 80 companies as a function of score is clearly unbalanced. Twenty-one companies fall in the range (50,60), which is higher than the other score ranges. None of the companies fall within the range (90,100). If the dividing point is set at a score of 60, the lower scores (≤60) account for 58 companies, with the rest having scores >60.

### 4.2. Attribute Reduction

The domain U contains the 80 companies in this study. The granularity score is incremented by 10, forming the decision-attribute set D. We then construct the individual factors that may affect the granularity grade, which form the condition-attribute set C (C_1_: expected revenue; C_2_: certification system, C_3_: SC integration degree; C_4_: manager education level; C_5_: company sales; C_6_: TS operation staff number; C_7_: information management level; C_8_: cognition of TS; C_9_: system maintenance investment). The initial decision table (partial) is presented in Table 4.

According to Table 4, the condition-attribute set and decision-attribute set are classified according to the rule of merging the same attributes. Upon deleting one condition attribute, the other condition attributes are classified. The classification result is shown below:

(4) $$U/IND\left(C-C_{2}\right)\neq U/IND\left(C\right)$$

(5) $$U/IND\left(C-C_{3}\right)\neq U/IND\left(C\right)$$

(6) $$U/IND\left(C-C_{4}\right)\neq U/IND\left(C\right)$$

(7) $$U/IND\left(C-C_{5}\right)\neq U/IND\left(C\right)$$

(8) $$U/IND\left(C-C_{6}\right)=U/IND\left(C\right)$$

(9) $$U/IND\left(C-C_{7}\right)\neq U/IND\left(C\right)$$

(10) $$U/IND\left(C-C_{8}\right)\neq U/IND\left(C\right)$$

(11) $$U/IND\left(C-C_{9}\right)\neq U/IND\left(C\right)$$

If $U/IND\left(C-C_{i}\right)=U/IND\left(C\right)$, attribute *C_i_* can be reduced. Therefore, the unimportant condition attribute C6 is deleted, and the condition attributes after reduction are C_1_–C_5_, C_7_–C_9_. 

### 4.3. Analysis of Importance of Attributes

To determine the importance of a given condition attribute, the classification of the domain relative to the decision attribute is analyzed after removing the given condition attribute. For example, POS_C_(D), which is an attribute in U/IND(D), is compared with the classification in U/IND(C). If the attributes in U/IND(D) exist in the same class as in U/IND(C), the attribute remains; if not, the attribute is deleted [45]. We list the POS values for various conditions in Table 5.

The degree of importance is calculated as per Section 3.4.4 Attribute importance. The original degree of importance and normalized values are listed in Table 5. In terms of degree of importance, the attributes are: C_1_(expected revenue) > C_3_(SC integration degree) > C_8_(cognition of TS) > C_2_(certification system) > C_5_(company sales) > C_7_(information management level) > C_9_(system maintenance investment) > C_4_(manager education level). Expected revenue is the most important factor that affects traceability granularity. The companies that adopted higher-granularity TS expected to obtain more revenue. Implementation of the TS relies on supply-chain cooperation. With the intensive food-safety requirement for customers, enhancing TS cognition plays a significant role in promoting the adoption of a higher-granularity TS. Thus, a certification system is not only the basis for implementing TS but also for its improvement. Company sales and system maintenance determine sustainability. Manager education level is the smallest factor, perhaps because of the diverse levels of information acquisition among managers.

## 5. Main Conclusions and Implications

Traceability granularity is an effective method for evaluating TS levels. This study uses 80 vegetable companies from Tianjin city as examples and calculates their granularity scores by using the traceability-granularity evaluation model and information collected from the TS platform. The results show that the companies are unequally distributed as a function of score. The score range (50,60) contains the most companies (21). Furthermore, the factors and their level of importance in influencing traceability granularity are analyzed by adopting the rough set method based on nine preselected factors. The results of the analysis show that the factor “number of TS operation staff” is deleted because it is unimportant. The other factors are ranked from most important to least important as follows: expected revenue > SC integration degree > cognition of TS > certification system > company sales > information management level > system maintenance investment > manager education level. The research results can help identify which enterprises do well in traceability granularity. The importance of influencing factors can be used as a reference for enterprises to further improve and will help to implement an effective traceability system. At the same time, it can be used as an important reference for the government to evaluate the effect of implementing a traceability system in enterprises, which is helpful to improve the supervision of enterprises by government departments and ensure food quality and safety.

Based on the above research conclusions, the corresponding implications are as follows: First, the market mechanism of high prices and high quality should be established. The higher traceability granularity implies more safety and trustworthy information and a higher input cost. Ensuring income is the basis of company investment decisions. Second, government investment in constructing the TS should be increased. In the early stages of establishing the TS, relying solely on the spontaneous behavior of companies is insufficient. The government should formulate fiscal and taxation policies to encourage companies to establish a TS. Meanwhile, some basic traceability requirements should be put forward by the government through laws and regulations. Third, the degree of organization of SC companies should be enhanced. The upstream and downstream cooperation mechanism may be established with the core of large-scale agro-food production and processing companies. Thereby, the degree of organization of production and management should improve, and the construction of the TS should advance.

## Figures and Tables

**Table 1:** Two-layer index, index weight, description, and quantization scores.

First Layer Indexes	Second Layer Indicators	Weight	Indicators Description	Scores
Precision	External trace unit		Single product	5
		0.1985	Single batch	3
			Mixed batch	1
	Internal flow unit		Single product	5
		0.1141	Single batch	3
			Mixed batch	1
	IU conversion		One-to-one	5
		0.0872	One-to-many	4
			Many-to-one	2
			Many-to-many	1
Breadth	Information collection content	0.1870	Basic information, forward source information, backward direction information, process information	5
			All information except process information	4
			Basic information, forward source information or backward direction information	3
			only basic information	1
	Information update frequency	0.1248	Hourly level	5
			Daily level	3
			Monthly level	1
Depth	Backward tracing distance		Tracking more than 3 levels	5 (at the front of the supply chain is default to 5)
		0.1442	Tracking 2 levels	4
			Tracking 1 level	3
			Tracking less than 1 level	1
	Forward tracking distance		Tracking more than 3 levels	5 (at the end of the supply chain is default to 5)
		0.1442	Tracking 2 levels	4
			Tracking 1 level	3
			Tracking less than 1 level	1

**Table 2:** Main factors that affect enterprise motivation on TS.

Factors Type	Influencing Factors	Mainly Literature
External factors	Expected revenue	[36,37]
	Government policy	[26,38,39]
	Market requirement	[17,40]
Internal factors	Certification system	[41]
	SC integration degree	[42]
	Manager education level	[40]
	Enterprise turnover	[43]
	TS operation staff ratio	[44]
	Informationization management level	[45]
	Cognition of TS	[46,47]
	System maintenance investment	[48]

**Table 3:** Characteristics of surveyed enterprises in Tianjin city.

Influencing Factors	Factors Feature	Enterprise Number	Percentage (%)
Expected revenue	<0%	5	6.25
	0–5%	24	30
	5–10%	37	46.25
	10–20%	12	15
	>20%	2	2.5
Certification system	No certification	0	0
	ISO 9000 certification	12	15
	GAP/HACCP	10	12.5
	Other certification	43	53.75
	ISO9000/GAP/HACCP + other certification	15	18.75
SC integration degree	Very low	16	20
	Low	38	47.5
	Medium	12	15
	High	9	11.25
	Very high	5	6.25
Manager education level	Less than senior high school	4	5
	Senior high school	11	13.75
	College level	48	60
	Master degree	15	18.75
	Doctor degree	2	2.5
Enterprise turnover	<5 million Yuan	21	26.25
	5–10 million Yuan	24	30
	10–30 million Yuan	18	22.5
	30–50 million	14	17.5
	>50 million	3	3.75
TS operation staff	No special operation staff	12	15
	One person	36	45
	Two persons	22	27.5
	3–5 persons	8	10
	>5 persons	2	2.5
Information management level	Very low	8	10
	Low	22	27.5
	Medium	32	40
	High	13	16.25
	Very high	5	6.25
Cognition of TS	Very low	4	5
	Low	9	11.25
	Medium	40	50
	High	21	26.25
	Very high	6	7.5
System maintenance investment	0%	2	2.5
	0–1%	14	17.5
	1–3%	42	52.5
	3–5%	19	23.75
	>5%	3	3.75

**Table 4:** Initial decision table of TS granularity influencing factors (partial).

U		1	2	3	…	78	79	80
**C**	**C1 (Expected revenue)**	2	2	4		1	3	3
	**C2 (Certification system)**	1	3	2		5	2	2
	**C3 (SC integration degree)**	3	1	3		3	2	3
	**C4 (Manager education level)**	2	1	4		4	1	5
	**C5 (Company sales)**	1	1	2		2	4	3
	**C6 (TS operation staff number)**	1	2	5		1	3	2
	**C7 (Information management level)**	2	2	3		2	5	1
	**C8 (Cognition of TS)**	4	1	4		5	1	2
	**C9 (System maintenance investment)**	2	3	5		1	1	4
**D**		2	1	4		2	4	3

**Table 5:** POS value and importance degree.

POS Assortment	Assortment Number	Importance Degree	Normalized Importance Degree
POS_C_(D)	58		
POS_C-C1_(D)	54	0.931	0.205
POS_C-C2_(D)	33	0.569	0.126
POS_C-C3_(D)	48	0.828	0.182
POS_C-C4_(D)	15	0.259	0.057
POS_C-C5_(D)	30	0.517	0.114
POS_C-C7_(D)	25	0.431	0.095
POS_C-C8_(D)	41	0.707	0.156
POS_C-C9_(D)	17	0.293	0.065

## Data Availability

No new data were created or analyzed in this study. Data sharing is not applicable to this article.
